# Author Correction: Specific CD4^+^ T cell phenotypes associate with bacterial control in people who ‘resist’ infection with *Mycobacterium tuberculosis*

**DOI:** 10.1038/s41590-024-01959-x

**Published:** 2024-08-19

**Authors:** Meng Sun, Jolie M. Phan, Nathan S. Kieswetter, Huang Huang, Krystle K. Q. Yu, Malisa T. Smith, Yiran E. Liu, Chuangqi Wang, Sanjana Gupta, Gerlinde Obermoser, Holden Terry Maecker, Akshaya Krishnan, Sundari Suresh, Neha Gupta, Mary Rieck, Peter Acs, Mustafa Ghanizada, Shin-Heng Chiou, Purvesh Khatri, W. Henry Boom, Thomas R. Hawn, Catherine M. Stein, Harriet Mayanja-Kizza, Mark M. Davis, Chetan Seshadri

**Affiliations:** 1 Institute for Immunity, Transplantation and Infection, School of Medicine, Stanford University, Stanford, CA USA; 2 Department of Medicine, University of Washington School of Medicine, Seattle, WA USA; 3 Department of Epidemiology and Population Health, School of Medicine, Stanford University, Stanford, CA USA; 4 Department of Immunology and Microbiology, University of Colorado, Anschutz Medicine Campus, Aurora, CO USA; 5 Center for Biomedical Informatics Research, Department of Medicine, Stanford University, Stanford, CA USA; 6 Human Immune Monitoring Center, Institute for Immunity, Transplantation and Infection, School of Medicine, Stanford University, Stanford, CA USA; 7 Department of Immunology and Microbiology, Faculty of Health and Medical Sciences, University of Copenhagen, Copenhagen, Denmark; 8 Division of Medical Oncology, Rutgers Cancer Institute of New Jersey, Department of Medicine, Rutgers Robert Wood Johnson Medical School, Rutgers University, New Brunswick, NJ USA; 9 Department of Biomedical Data Sciences, School of Medicine, Stanford University, Stanford, CA USA; 10 Department of Medicine, Case Western Reserve University, Cleveland, OH USA; 11 Department of Population and Quantitative Health Sciences, Case Western Reserve University, Cleveland, OH USA; 12 Department of Medicine, Makerere University School of Medicine, Kampala, Uganda; 13 Department of Microbiology and Immunology, Stanford University School of Medicine, Stanford, CA USA; 14 Howard Hughes Medical Institute, Stanford University School of Medicine, Stanford, CA USA

**Keywords:** Tuberculosis, Cellular immunity

Correction to: *Nature Immunology* 10.1038/s41590-024-01897-8. Published online 12 July 2024.

In the version of this article initially published, the name of Chuangqi Wang was misspelled (“Chuanqi”); in Fig. 2a, data were missing in the lower-right plot and in Fig. 2f, the labels, now reading “LTBI” and “RSTR” in top-down fashion, were reversed. The changes have been made in the HTML and PDF versions of the article.

