# Supplementary material for: Specific CD4+ T cell phenotypes associate with bacterial control in people who ‘resist’ infection with Mycobacterium tuberculosis
Source: Nat Immunol. 2024 Jul 12;25(8):1411–21. doi: 10.1038/s41590-024-01897-8 (PMC11291275; doi:10.1038/s41590-024-01897-8)
Supplement: Supplementary file 1 — Reporting Summary [file 41590_2024_1897_MOESM1_ESM.pdf]

nature portfolio

Corresponding author(s): Chetan Seshadri

Last updated by author(s): Jun 11, 2024

Reporting Summary

Nature Portfolio wishes to improve the reproducibility of the work that we publish. This form provides structure for consistency and transparency in reporting. For further information on Nature Portfolio policies, see our Editorial Policies and the Editorial Policy Checklist.

Statistics

For all statistical analyses, confirm that the following items are present in the figure legend, table legend, main text, or Methods section.

n/a	Confirmed	
☐	☒	The exact sample size (*n*) for each experimental group/condition, given as a discrete number and unit of measurement
☐	☒	A statement on whether measurements were taken from distinct samples or whether the same sample was measured repeatedly
☐	☒	The statistical test(s) used AND whether they are one- or two-sided<br>*Only common tests should be described solely by name; describe more complex techniques in the Methods section.*
☒	☐	A description of all covariates tested
☐	☒	A description of any assumptions or corrections, such as tests of normality and adjustment for multiple comparisons
☐	☒	A full description of the statistical parameters including central tendency (e.g. means) or other basic estimates (e.g. regression coefficient) AND variation (e.g. standard deviation) or associated estimates of uncertainty (e.g. confidence intervals)
☐	☒	For null hypothesis testing, the test statistic (e.g. *F*, *t*, *r*) with confidence intervals, effect sizes, degrees of freedom and *P* value noted<br>*Give P values as exact values whenever suitable.*
☒	☐	For Bayesian analysis, information on the choice of priors and Markov chain Monte Carlo settings
☒	☐	For hierarchical and complex designs, identification of the appropriate level for tests and full reporting of outcomes
☒	☐	Estimates of effect sizes (e.g. Cohen's *d*, Pearson's *r*), indicating how they were calculated

Our web collection on statistics for biologists contains articles on many of the points above.

Software and code

Policy information about availability of computer code

Data collection	The index-sorting data were collected using BD FACSDiva 8.0.1. Single-cell sequencing used Illumina instrumentation and softwares described in the method session. The flow cytometry data in the validation study were collected using a BD LSRFortessa. The multiplex cytokine data were acquired using the Bio-Plex 200 suspension array system (Bio-Rad, Hercules, CA). The ELISA plates were read at 450 nm using a CLARIOstar Plus Microplate Reader (BMG LabTech, Ortenberg, Germany).

Data analysis	The index-sort and targeted PCR data in SELECT-seq were analyzed in CATALYST package (V2.1.11) in R (V4.1.2). For scRNA-seq in SELECT-seq, the raw data was first processed in Python (V3.9) and then analyzed using the Seurat package (V4) in R (V4.1.2). For the network analysis, we screened the immune-related genes (GO0002376:immune_system_process) and ran the STRINGdb (V2.16.4) network clustering algorithm. The gene regulatory network inference and motif discovery were conducted using SCENIC (V1.1.2). Code to complete SELECT-seq analysis and analyses of ACS and NHP cohort data can be found at https://github.com/ttsunmeng/TB_RSTR_ESAT6CFP10_SelectSeq_analysis_pipeline.<br><br>The flow cytometry data were compensated and gated using FlowJo (v9.9.6) (BD Biosciences, San Jose, CA). Representative gating trees of the low exposure cohort and the household contact cohort are shown in Extended Data Fig. 1, 2, 4, and 6. The data were then processed using the OpenCyto framework (V2.16.1) in the R programming environment (V4.1.2). With the data from the endemic controls, Combinatorial Polyfunctionality Analysis of Antigen-Specific T Cell Subsets (COMPASS) (V1.19.4) was used to achieve a comprehensive and unbiased analysis of the activation profiles of antigen-specific T cells. The R package ComplexHeatmap (V1.15.1) was used to visualize COMPASS posterior probabilities of response. The multiplex cytokine and TGFβ ELISA data were exported as CSV files from the Bio-Plex 200 CLARIOstar Plus Microplate Reader, respectively. All the cytokine data were merged, cleaned, and analyzed in R. The code to complete multiplex cytokine and flow cytometry analyses can be found at https://github.com/seshadrilab/cd4-phenotypes-sun-2024-procartaplex and https://github.com/seshadrilab/cd4-phenotypes-sun-2024-flow.

For manuscripts utilizing custom algorithms or software that are central to the research but not yet described in published literature, software must be made available to editors and reviewers. We strongly encourage code deposition in a community repository (e.g. GitHub). See the Nature Portfolio guidelines for submitting code & software for further information.

Data

Policy information about availability of data

All manuscripts must include a data availability statement. This statement should provide the following information, where applicable:

- Accession codes, unique identifiers, or web links for publicly available datasets
- A description of any restrictions on data availability
- For clinical datasets or third party data, please ensure that the statement adheres to our policy

All the validation flow cytometry data are available for download from ImmPort at https://www.immport.org under study accession number SDY2277 and at Fairdomhub at https://fairdomhub.org/studies/1179. The processed Seurat object generated from SELECT-Seq data is available at Zenodo at https://zenodo.org/records/7946277. The raw and processed SELECT-Seq data is available at Gene Expression Omnibus (GEO; accession number GSE267774). The gene sets GO:0072539 and GO:0002376 from MSigDB were used to analyze SELECT-Seq data. From the ACS cohort, whole blood bulk transcriptomics data is available at GEO (accession number GSE79362) from Zak et al. 2016 and single-cell targeted transcriptomics data can be found in supplementary materials in Musvosvi et al. 2023.

Human research participants

Policy information about studies involving human research participants and Sex and Gender in Research.

Reporting on sex and gender	Samples from study participants were selected after matching for sex. In the low exposure control cohort, subjects included in this study consisted of 19 male subjects and 17 female subjects (Supplementary Table 2). In the household contact cohort, subjects included in this study consisted of 38 male subjects and 39 female subjects (Supplementary Tables 4-5). We analyzed 524 T cells from the SELECT-Seq data after quality control filtering and found that the major axes of transcriptomic variance were group assignment (LTBI or RSTR) and sex, which were not mutually exclusive (Extended Data Fig. 3).
Population characteristics	For the low exposure cohort, the median age of the concordant positive (LTBI) group was 23 years and the median age of the concordant negative (TST-/IGRA-) group was 22 years. The participants of the LTBI and TST-/IGRA- group were 52.9% male and 52.6% male, respectively. All of the participants were HIV negative.<br><br>For the household contact cohort, the median age of the RSTR group was 23.2 years and the median age of the LTBI group was 24.8 years. The participants of the RSTR and LTBI groups were 48.6% male and 50% male, respectively. All of the participants were HIV negative.
Recruitment	The low exposure cohort was enrolled from a low TB incidence district that was identified by the Kampala Capital City Authority based on low TB transmission rates. Subjects were screened and enrolled for health assessment and blood draws between 2017 and 2018 in Uganda (Extended Data Fig. 1). All healthy, non-pregnant participants were eligible. A total of 247 individuals were approached in this district, of which 230 consented and were screened for previous TB treatment, pregnancy, medications, and serious illnesses. A total of 220 healthy, non-pregnant individuals were enrolled, and 211 of the enrolled were found to be non-infected with HIV. All participants reported no known contact with a TB case.<br><br>As we have previously described (Stein et al. 2018), household contacts of sputum culture positive cases of pulmonary TB were enrolled between 2002 and 2012 as part of the Kawempe Community Health Study. Adults with pulmonary TB were recruited from clinics at the Uganda National TB and Leprosy Program treatment center at Mulago Hospital, referred to the TB research clinic at Mulago Hospital, or recruited through community sensitization efforts in the Kawempe division of Kampala. Selection bias is primarily driven by referral to clinical care for which an effort was made to recruit through community sensitization. At baseline, individuals had no active Mtb infection determined by sputum culture and radiology. Upon enrollment, individuals were longitudinally screened during a two-year follow-up period by TST (Mantoux method, 0.1 ml of 5 tuberculin units of purified protein derivative (PPD), Tubersol; Connaught Laboratories), in which a positive TST was defined as an induration of >10 mm for individuals non-infected with HIV and >5mm for individuals infected with HIV. In this initial study, a total of 2,585 individuals were enrolled in the household contact cohort. Of these individuals, 198 (10.7%) remained persistently TST negative over the two-year follow-up period upon their enrollment. Between 2014 and 2017, 691 household contacts from the initial study were identified as eligible for retracing according to the epidemiologic risk score criteria previously published. Of these individuals, 441 (63.8%) were enrolled in a subsequent longitudinal follow-up retracing

	study. The mean time between enrollment in the initial study and completion of the retracing study was 9.5 years. During the retracing study, individuals completed three QFT assays over two years. On their final visit, individuals also underwent the TST (positive TST defined above). Resisters (RSTR) were classified as such if all TST assays (five from the initial study and one at the end of the retracing study) and the three QFTs from the retracing study were concordantly negative, while latent TB (LTBI) participants were classified as such if all TST and QFT assays were positive. All study subjects gave written, informed consent, approved by the National AIDS Research Committee, the Uganda National Council for Science and Technology, and the institutional review board at University Hospitals Cleveland Medical Center.
Ethics oversight	The household contact retracing study protocol was reviewed and approved by the National AIDS Research Committee, The Uganda National Council on Science and Technology, and the institutional review board at University Hospitals Cleveland Medical Center.

Note that full information on the approval of the study protocol must also be provided in the manuscript.

Field-specific reporting

Please select the one below that is the best fit for your research. If you are not sure, read the appropriate sections before making your selection.

☒ Life sciences ☐ Behavioural & social sciences ☐ Ecological, evolutionary & environmental sciences

For a reference copy of the document with all sections, see nature.com/documents/nr-reporting-summary-flat.pdf

Life sciences study design

All studies must disclose on these points even when the disclosure is negative.

Sample size	No power calculations were performed to pre-determine sample sizes, but our sample sizes are similar to those reported in our published studies of this cohort (Lu et al. 2019, Simmons et al. 2021).
Data exclusions	In the validation flow cytometry study, PBMC from two RSTR subjects were found to have bacterial contamination after overnight rest and were excluded from data acquisition and analysis.
Replication	Verification of reproducibility of the low exposure control cohort findings could not be performed because samples from an independent cohort of LTBI and TST-/IGRA- subjects from the low exposure control cohort were not available. Verification of SELECT-Seq findings of the household contact cohort was performed by validation flow cytometry experiments with an independent cohort of RSTR and LTBI subjects from the household contact cohort.
Randomization	Participant allocation was not randomized nor applicable to the study. PBMC from a subset of LTBI and RSTR subjects were selected after matching for age, sex, exposure risk score, and documented lack of HIV co-infection.
Blinding	The investigators were blinded to group allocation during acquisition of flow cytometry data in the validation household contact cohort.

Reporting for specific materials, systems and methods

We require information from authors about some types of materials, experimental systems and methods used in many studies. Here, indicate whether each material, system or method listed is relevant to your study. If you are not sure if a list item applies to your research, read the appropriate section before selecting a response.

Materials & experimental systems

n/a	Involved in the study	
☐	☒	Antibodies
☒	☐	Eukaryotic cell lines
☒	☐	Palaeontology and archaeology
☒	☐	Animals and other organisms
☒	☐	Clinical data
☒	☐	Dual use research of concern

Methods

n/a	Involved in the study	
☒	☐	ChIP-seq
☐	☒	Flow cytometry
☒	☐	MRI-based neuroimaging

Antibodies

Antibodies used	The following antibody information is also reported in Supplementary Table 3, along with their titers.<br><br>The ICS results in Fig. 1 utilized the "IPEC" panel which consisted of the following antibodies/markers: anti-CD107a PE-Cy7 (clone H4A3, BD Biosciences, catalog no. 561348); anti-CD3 ECD (clone UCHT1, Beckman Coulter, catalog no. IM2705U); anti-CD4 APC-Cy7 (clone 13B8.2, Beckman Coulter, catalog no. A94685); anti-CD8 PerCP-Cy5.5 (clone SK1, BD Biosciences, catalog no. 341051); anti-IL-2 PE (clone MQ1-17H12, BD Biosciences, catalog no. 559334); anti-IL-4 APC (clone MP4-25D2, BD Biosciences, catalog no. 554486);

anti-IL-5 APC (clone TRFK5, BioLegend, catalog no. 504306); anti-IL-13 APC (clone JES10-5A2, BioLegend, catalog no. 501907); anti-IFN-g V450 (clone B27, BD Biosciences, catalog no. 560371); anti-TNFa FITC (clone MAb11, BD Biosciences, catalog no. 554512); anti-IL-17A AF700 (clone BL168, BioLegend, catalog no. 512318); anti-CD154 PE-Cy5 (clone TRAP1, BD Biosciences, catalog no. 555701); LIVE/DEAD™ Fixable Aqua Dead Cell Stain (Invitrogen, catalog no. L34966).

The index-sort results in Fig. 2 utilized the "Index sort" panel which consisted of the following antibodies/markers: LIVE/DEAD™ Fixable Aqua Dead Cell Stain (Invitrogen, catalog no. L34966); anti-CD3 BV786 (clone UCHT1, BioLegend, catalog no. 300472); anti-CD4 BV605 (clone RPA-T4, BioLegend, catalog no. 300556); anti-CD8a BUV496 (clone RPA-T8, BD Biosciences, catalog no. 564804); anti-TCRab PE-Cy7 (clone IP26, BioLegend, catalog no. 306720); anti-CD14 BV510 (clone M5E2, BioLegend, catalog no. 301842); anti-CD19 BV510 (clone HIB19, BioLegend, catalog no. 302242); anti-CD16 PE-Cy5 (clone 3G8, BioLegend, catalog no. 302010); anti-CD45RA FITC (clone HI100, BioLegend, catalog no. 304106); anti-CD154 PE (clone TRAP1, BD Biosciences, catalog no. 555700); anti-CD137 APC-Fire 750 (clone 4B4-1, BioLegend, catalog no. 309834); anti-HLA-DR AF700 (clone LN3, BioLegend, catalog no. 327014); anti-CD38 BV711 (clone HIT2, BioLegend, catalog no. 303528); anti-CD69 BUV396 (clone FN50, BD Biosciences, catalog no. 564364); anti-CD127 BUV737 (clone HIL-7R-M21, BD Biosciences, catalog no. 564300); anti-CXCR3 AF647 (clone G025H7, BioLegend, catalog no. 353712); anti-CCR6 BV421 (clone G034E3, BioLegend, catalog no. 353408); anti-CD25 PE-Dazzle 594 (clone M-A251, BioLegend, catalog no. 356126).

The ICS results in Figs. 3 and 5 utilized the "Th" panel which consisted of the following antibodies/markers: Zombie Yellow Fixable Viability Kit (BioLegend, catalog no. 423103); anti-CD3 BUV395 (clone UCHT1, BD Biosciences, catalog no. 563546); anti-CD4 BB515 (clone L200, BD Biosciences, catalog no. 564419); anti-CD8a BV510 (clone RPA-T8, BD Biosciences, catalog no. 563256); anti-CD14 BV785 (clone M5E2, BioLegend, catalog no. 301840); anti-CD19 BV785 (clone SJ25C1, BioLegend, catalog no. 363028); anti-CD45RA BUV737 (clone HI100, BD Biosciences, catalog no. 612846); anti-CCR7 BV711 (clone 150503, BD Biosciences, catalog no. 566602); anti-CD154 PE-Cy5 (clone TRAP1, BD Biosciences, catalog no. 310802); anti-CD137 BV605 (clone 4B4-1, BioLegend, catalog no. 309821); anti-OX40 PE-Cy7 (clone ACT35, BioLegend, catalog no. 350012); anti-CTLA-4 BB700 (clone BNI3, BD Biosciences, catalog no. 566901); anti-CXCR3 PE-Dazzle 594 (clone G025H7, BioLegend, catalog no. 353735); anti-CCR6 APC-Cy7 (clone G034E3, BioLegend, catalog no. 353431); anti-T-bet PE (clone 4B10, BioLegend, catalog no. 644809); anti-RORγT AF647 (clone Q21-559, BD Biosciences, catalog no. 563620); anti-IFN-g V450 (clone B27, BD Biosciences, catalog no. 560371); anti-IL-17a AF700 (clone BL168, BioLegend, catalog no. 512318).

This ICS results in Fig. 4 utilized the "Treg" panel which consisted of the following antibodies/markers: Zombie Yellow Fixable Viability Kit (BioLegend, catalog no. 423103); anti-CD3 BUV395 (clone UCHT1, BD Biosciences, catalog no. 563546); anti-CD4 BB515 (clone L200, BD Biosciences, catalog no. 564419); anti-CD8a BV510 (clone RPA-T8, BD Biosciences, catalog no. 563256); anti-CD14 BV785 (clone M5E2, BioLegend, catalog no. 301840); anti-CD19 BV785 (clone SJ25C1, BioLegend, catalog no. 363028); anti-CCR7 BV711 (clone 150503, BD Biosciences, catalog no. 566602); anti-CD154 PE-Cy5 (clone TRAP1, BD Biosciences, catalog no. 310802); anti-CD137 BV605 (clone 4B4-1, BioLegend, catalog no. 309821); anti-OX40 PE-Cy7 (clone ACT35, BioLegend, catalog no. 350012); anti-CTLA-4 BB700 (clone BNI3, BD Biosciences, catalog no. 566901); anti-FoxP3 eFluor 660 (clone PCH101, eBiosciences, catalog no. 50-4776-41); anti-CD25 BV421 (clone 2A3, BD Biosciences, catalog no. 612813); anti-CD39 APC-Cy7 (clone A1, BioLegend, catalog no. 328225); anti-CD73 BUV737 (clone AD2, BD Biosciences, catalog no. 612813); anti-IL-10 PE (clone JES3-19F1, BD Biosciences, catalog no. 559330).

The Tscm results in Extended Data Fig. 5 utilized the "Tscm" panel which consisted of the following antibodies/markers: anti-CD45RO PerCP Cy5.5 (clone UCHL1, BioLegend, catalog no. 304222); anti-CD45RA PE Cy7 (clone HI100, BioLegend, catalog no. 304126); anti-CCR7 PE (clone G043H7, BioLegend, catalog no. 353204); anti-CD95 PE-Dazzle 594 (clone DX2, BD Biosciences, catalog no. 305633); anti-CD154 BV711 (clone 24-31, BioLegend, catalog no. 310837); anti-CD69 BV421 (clone FN50, BioLegend, catalog no. 310930); anti-CD4 APC-H7 (clone L200, BD Biosciences, catalog no. 560837); anti-TCRa/b APC (clone IP26, BioLegend, catalog no. 306718); anti-CD62L BV785 (clone DREG-56, BioLegend, catalog no. 304830); anti-CD8 BV650 (clone RPA-T8, BioLegend, catalog no. 301042); anti-CD3 BV421 (clone SP34-2, BD Biosciences, catalog no. 562877); anti-CD95 BUV395 (clone DX2, BD Biosciences, catalog no. BDB740306); LIVE/DEAD™ Fixable Aqua Dead Cell Stain (ThermoFisher, catalog no. L34957); BioTracker 488 Green CSFE (SigmaAldrich, catalog no. SCT110)

Validation

All antibodies were purchased from commercial suppliers (BD Biosciences, BioLegend, Invitrogen, and Beckman Coulter). The manufacturers state that these antibodies are research use only (ROU) and have been tested for flow cytometry application using human samples. Fluorescence minus one experiments were performed and and antibody titers were validated experimentally prior to performing the flow cytometry experiments in this study to determine the optimal staining volumes.. Gating strategies can be found in Extended Data Fig. 1, 2, 4, and 6.

Flow Cytometry

Plots

Confirm that:

☒ The axis labels state the marker and fluorochrome used (e.g. CD4-FITC).

☒ The axis scales are clearly visible. Include numbers along axes only for bottom left plot of group (a 'group' is an analysis of identical markers).

☒ All plots are contour plots with outliers or pseudocolor plots.

☒ A numerical value for number of cells or percentage (with statistics) is provided.

Methodology

Sample preparation

Human peripheral blood mononuclear cells (PBMCs) were isolated from whole blood by Ficoll-Hypaque density centrifugation and cryopreserved until use. Cryopreserved PBMC were thawed in warm, sterile-filtered RPMI 1640 (Gibco, Waltham, MA) supplemented with 10% fetal bovine serum (FBS) (HyClone, Logan, UT) and 2 µL/mL Benzonase (Millipore,

Burlington, MA). Centrifugation was performed at 300xg for 10 minutes. The cells were enumerated using the Guava easyCyte (Millipore, Burlington, MA) with guavaSoft 2.6 software and centrifuged again at 300xg for 10 minutes. The cells were then resuspended in a 50 mL conical at a density of 2 × 106 cells/394 mL in RPMI/10% FBS with caps loosely secured and allowed to rest overnight at 37°C/5% CO2. The following day, the cells were enumerated using the Guava easyCyte and resuspended at a density of 5 × 106 cells/mL. To observe ICS following antigen stimulation, approximately 1 × 106 cells/well were plated into a 96-well U-bottom plate and stimulated in the presence of ESAT-6/CFP-10 peptide pool (final concentration of 1 µg/mL of each peptide, BEI Resources), 100 µg ml–1 M.tb whole cell lysate (H37Rv, BEI Resources), DMSO (0.5% for the endemic controls and 0.18% for the household contacts) (Sigma, St. Louis, MO). In addition to antigen, each stimulation cocktail consisted of 1 µg/mL anti-CD28/49d (BD Biosciences, San Jose, CA), 10 µg/mL Brefeldin A (Sigma, St. Louis, MO), and GolgiStop (BD Biosciences, San Jose, CA) prepared according to manufacturer's instructions was added to each sample. Cells were incubated at 37oC/5% CO2. EDTA (Thermo Fisher Scientific, Waltham, MA) was then added to disaggregate cells at a final concentration of 2 mM. Samples were stored overnight at 4C and stained the following day.

Instrument

A BD LSRFortessa was used to acquire the ICS data, equipped with a high-throughput sampler and configured with blue (488 nm), green (532 nm), red (628 nm), violet (405 nm) and ultraviolet (355 nm) lasers using standardized good clinical laboratory practice procedures to minimize the variability of data generated. The index-sorting data were collected using BD FACSDiva 8.0.1.

Software

The index-sort data were analyzed using the CATALYST package in R (v4.2.1). ICS data were compensated and gated using FlowJo (v9.9.6) (BD Biosciences, San Jose, CA). Representative gating trees of the low exposure cohort and the household contact cohort are shown in Extended Data Fig. 1, 2, 4, and 6. The data were then processed using the OpenCyto framework in the R programming environment. With the data from the endemic controls, Combinatorial Polyfunctionality Analysis of Antigen-Specific T Cell Subsets (COMPASS) was used to achieve a comprehensive and unbiased analysis of the activation profiles of antigen-specific T cells. The code to complete the validation flow cytometry analyses can be found at https://github.com/seshadrilab/rstr-ins-validation.

Cell population abundance

In the index-sort data, the median frequencies of activated (CD69+/CD154+ and CD69+/CD137+) T cells in RSTR and LTBI samples were 0.30% (range 0.21% - 0.35%) and 0.46% (range 0.27% - 0.89%), respectively.

Gating strategy

Flow cytometry gating strategies can be seen in Extended Data Fig. 1-2 and 4-6.

For the low exposure cohort ICS experiments, gating began with viable CD3+ cells to identify T cells, followed by a singlet gate (FSC-A vs. FSC-H) and an IL-4/5/13 keeper gate to clean up events. CD4 T cells were identified, and cytokine and activation marker expressions were visualized by gating against IFN-γ.

For the index-sort in the household contact cohort, a lymphocyte gate (FSC-A vs. SSC-A) and a singlet gate (FSC-A vs. FSC-H) were applied. T cells were identified as CD3+/CD14-/CD19-. T cells positive for the TCRαβ marker were gated on and separated into CD4 or CD8 subsets. Activated cells were gated on using CD69, and sorting of activated CD4 and CD8 T cells was done using CD137 and CD154 markers, respectively. Gating of activation and functional markers were determined by DMSO controls.

For the Treg panel in the validation ICS experiments, a time gate (Time vs. FSC-A) was applied to exclude events affected by sample acquisition aberrations, followed by T cell identification by lymphocyte size (FSC-A vs. SSC-A), CD3, CD14, and CD19 markers. A singlet gate (FSC-A vs. FSC-H) was then applied, followed by the identification of viable cells and a second lymphocyte gate (FSC-A vs. SSC-A). CD4 T cells were identified, and positive populations for Treg phenotypic, functional, and activation makers were determined by DMSO controls.

For the Th panel in the validation ICS experiments, the gating strategy from the time gate (Time vs. FSC-A) to the second lymphocyte gate (FSC-A vs. SSC-A) was the same as the Treg panel gating strategy. CD4 T cells were then identified, and positive populations for Th1 and Th17 phenotypic, functional, and activation makers were determined by DMSO controls. A secondary gate was applied to identify IL-17A+ T cells due to spillover spreading.

☒ Tick this box to confirm that a figure exemplifying the gating strategy is provided in the Supplementary Information.
